# Association of serum iron with all-cause mortality and cardiovascular mortality in the cardiovascular patients: a retrospective cohort study based on the NHANES 1999–2018

**DOI:** 10.3389/fcvm.2024.1414792

**Published:** 2024-12-10

**Authors:** Jing Lu, Zhen Ma, Xiaoxue Zhang, Wenhao Zhong, Yunzeng Zou, Jie Yuan

**Affiliations:** Shanghai Institute of Cardiovascular Diseases, Zhongshan Hospital and Institutes of Biomedical Sciences, Fudan University, Shanghai, China

**Keywords:** cardiovascular disease, serum iron, national health and nutrition examination survey (NHANES), all-cause mortality, cardiovascular mortality

## Abstract

**Background:**

Cardiovascular disease (CVD) is one of the leading global causes of death, and serum iron (SI) levels may be associated with the mortality of CVD. However, there is still a knowledge gap regarding the relationship between SI and mortality in the CVD population.

**Methods:**

An analysis was conducted utilizing data from the National Health and Nutrition Examination Survey (NHANES) from 1999 to 2018. In our study, SI was used as the independent variable, and the mortality of the CVD patients was considered as the outcome. Kaplan–Meier curves, multivariable Cox proportional hazards model, and restricted cubic spline were employed to examine the association between SI and all-cause mortality and cardiovascular mortality in CVD patients. Subgroup analysis was also carried out based on age, sex, weight, hypertension, Type 2 diabetes mellitus, and smoking status.

**Results:**

A retrospective cohort study design was utilized, incorporating data from 1,903 CVD patients with an average age of 64.29 years. Kaplan–Meier survival analysis demonstrated significant differences in all-cause mortality and cardiovascular mortality among the CVD patients based on quartiles of SI. Following multivariable adjustment, lower SI was associated with an increased risk of all-cause and cardiovascular mortality in CVD patients. The highest quartile of SI exhibited a 43% reduction in all-cause mortality (HR = 0.57, 95% CI: 0.45–0.72) and a 74% reduction in cardiovascular mortality (HR = 0.26, 95% CI: 0.16–0.43) when compared to the lowest quartile. Restricted cubic spline showed a nonlinear relationship between SI and all-cause mortality and a linear relationship between SI and cardiovascular mortality. Additionally, the inverse relationship between SI levels and outcomes in the CVD patients remained consistent in subgroup analysis.

**Conclusion:**

Higher SI is associated with a decreased risk of all-cause and cardiovascular mortality in CVD patients. Our results emphasize the importance of iron supplementation for this particular group.

## Introduction

Cardiovascular diseases (CVD), encompassing heart failure, coronary heart disease (CHD), angina, heart attack, and stroke, are recognized as the leading global cause of mortality (1). Over the past decade, the worldwide death toll from CVD has surged by 12.5%, now constituting approximately one-third of all global deaths (2). In 2019, an estimated 2.52 billion individuals were reported as CVD patients globally across 204 countries and regions, reflecting a decrease from 2.71 billion in 1990. Despite this decline, there was an alarming increase of 6.5 million deaths attributed to CVD, reaffirming its status as a primary global mortality contributor (3). Notably, advancing age is correlated with escalating mortality associated with CVD, presenting a substantial public health dilemma (4). Despite significant advancements in the diagnosis, treatment, and prevention of CVD over the past two decades, they persist as a formidable cause of mortality, posing a grave risk to human life.

In recent years, the significance of minerals in human physiological metabolism has gained widespread attention. Numerous studies have underscored the roles of magnesium, selenium, and calcium in CVD. Serum iron (SI), as an essential trace element, plays a pivotal role in anti-inflammatory processes and oxidative stress (5). Studies indicate that iron deficiency is prevalent in over 50% of outpatient heart failure cases (6). Furthermore, several clinical studies have illustrated that iron supplementation for heart failure patients with iron deficiency can substantially ameliorate symptoms (7–9). Additionally, SI has been linked to various clinical outcomes. Research on patients with acute ST-segment elevation myocardial infarction has demonstrated a connection between SI concentration and subsequent heart failure occurrence (10). Another study identified low SI as a prognostic risk factor for adverse outcomes in acute decompensated heart failure patients, independently of hemoglobin and ferritin levels. Moreover, SI has been associated with kidney function (11), fatty liver (1), tumors (12), and cognitive impairment (13). However, the exact relationship between SI levels and CVD remains ambiguous, and the prognosis of CVD patients based on SI remains uncertain.

In this study, utilizing data from the National Health and Nutrition Examination Survey (NHANES) in the United States, we investigate the association between SI and the mortality of CVD to gain further insights into the role of SI in CVD patients.

## Methods

### Data source

The NHANES is conducted by the National Center for Health Statistics (NCHS) and comprises a representative sample of non-institutionalized civilians in the United States. The study design employs a multi-stage probability sampling approach and gathers pertinent information including demographics, socioeconomic status, diet, and health status, among other factors. This study design has been sanctioned by the NCHS Institutional Review Board in adherence to the Helsinki Declaration. Before undergoing the examination, all participants provided informed consent. Moreover, we integrated data from the NHANES database with the National Death Index (NDI) to obtain relevant mortality details for each participant up to December 31, 2019. The cardiovascular mortality for individuals with CVD was ascertained utilizing ICD-10 disease codes (I00-I09, I11, I13, and I20-I51).

### Study population

**Figure 1:**
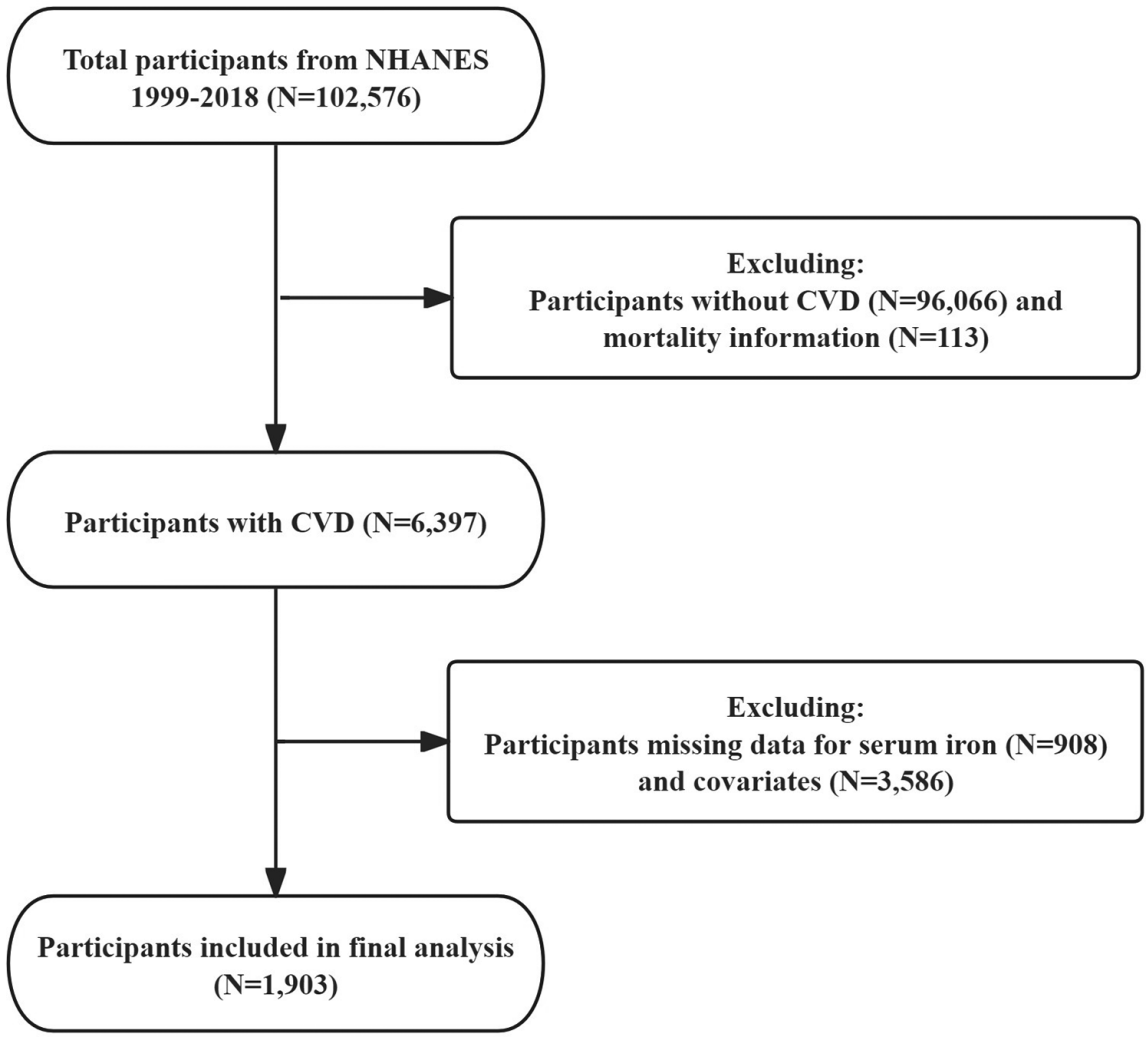
Flow chart of the study subjects.

For this study, we analyzed publicly available data from five 2-year cycles of NHANES (1999–2018). Inclusion criteria were as follows: participants diagnosed with CVD, with full mortality information, and possessing complete data on SI and covariates. Exclusions encompassed individuals without CVD or those with missing data related to CVD (*n* = 96,066), participants without mortality information (*n* = 113), individuals lacking SI data (*n* = 908), and those with missing covariate information (*n* = 3,586) (Figure 1). In addition, we compared the baseline characteristics of patients finally included in the study with the exclusion of participants with missing covariates (Supplementary Table 1).

### Outcome Variable

The diagnosis of CVD as the outcome variable was ascertained through self-reported physician diagnosis obtained during individual interviews utilizing standardized medical history questionnaires. Participants were asked, “Has a doctor or other health professional ever informed you of a diagnosis of congestive heart failure/coronary heart disease/myocardial infarction/stroke?” Any affirmative response to these questions categorized the individual as having cardiovascular disease.

### Expose Variable

SI was determined by electrochemiluminescence immunoassay and the units are expressed as umol/L.

### Other covariates

By existing literature, the following variables were considered as covariates: Demographic variables encompassed age, sex (male and female), race (Mexican American, non-Hispanic black, non-Hispanic white, other Hispanic, and other race), and poverty income ratio (PIR) was obtained from demographic data. Medical conditions such as hypertension and type 2 diabetes mellitus (DM) were determined by self-reported physician diagnoses provided by participants. Laboratory measurement variables comprised alanine aminotransferase (ALT), aspartate aminotransferase (AST), sodium, potassium, calcium, total cholesterol (TC), and high-density lipoprotein (HDL). Smoking and drinking were ascertained through questionnaires. For detailed insights on data collection, processing, quality assurance, and oversight, kindly refer to the NHANES website (https://www.cdc.gov/nchs/nhanes/index.htm).

### Statistical analysis

Participants in the study were stratified into four groups based on quartiles of SI (*Q*1 ≤ 11.10, 11.10 < *Q*2 ≤ 14.5, 14.5 < *Q*3 ≤ 18.40, and *Q*4 > 18.40, umol/L). All estimates were calculated accounting for sample weights. Weighted one-way analysis of variance and *t*-tests (for continuous variables) were employed, along with weighted chi-square tests (for categorical variables), to compare baseline characteristics. Kaplan–Meier (KM) curves were utilized to depict all-cause and cardiovascular survival for CVD patients. Weighted multivariable Cox regression analysis were conducted to individually investigate the relationship between SI both as a continuous variable and as a categorical variable with CVD patient all-cause and cardiovascular mortality. The multivariable Cox regression models were adjusted for pertinent variables based on the literature and clinical experience. Three models were developed in total: the Crude model solely included SI, Model 1 adjusted for age, sex, race, and PIR, and Model 2 additionally adjusted for drinking, smoking, BMI, hypertension, type 2 DM, ALT, AST, sodium, calcium, potassium, TC, and HDL. SI as a continuous variable underwent analysis using restricted cubic splines (RCS) to reveal the dose-response relationship with all-cause and cardiovascular mortality. Subgroup analyses were also conducted stratified by age, sex, BMI, hypertension, type 2 DM, drinking, and smoking.

All analyses were carried out using R software (version 4.1.3), and statistical significance was defined as *P* < 0.05.

## Results

### Baseline characteristics of participants

Table 1 presents a summary of the baseline characteristics of the 1,903 participants based on quartiles of SI, meeting the specified inclusion and exclusion criteria. The average age of participants was 64.29 years, with 55.36% being male. Significant variations were observed among the four groups in terms of sex, race, BMI, PIR, ALT, AST, calcium, TC, HDL, smoking, hypertension, cardiovascular mortality, and all-cause mortality (*P* < 0.05). The group with the highest quartile of SI exhibited a higher proportion of male participants, lower BMI, elevated PIR, higher ALT and AST levels, increased calcium, TC, and HDL levels, and was characterized by a higher representation of non-Hispanic white individuals, never-smokers, and individuals with hypertension, and has lower all-cause mortality, and cardiovascular mortality.

**Table 1:** Baseline characteristics of participants according to quartiles of SI.

	Total *N* = 1,903	*Q*1 *N* = 485	*Q*2 *N* = 483	*Q*3 *N* = 466	*Q*4 *N* = 469	*P* value
Age, years	64.29 (0.42)	62.65 (0.85)	65.13 (0.81)	64.07 (0.81)	65.24 (0.75)	0.104
Sex (%)						<0.001
Female	799 (44.64)	267 (63.37)	213 (46.24)	174 (36.12)	145 (33.05)	
Male	1,104 (55.36)	218 (36.63)	270 (53.76)	292 (63.88)	324 (66.95)	
Race (%)						0.016
Mexican American	212 (4.10)	37 (3.80)	52 (3.41)	60 (4.63)	63 (4.59)	
Non-Hispanic Black	373 (11.28)	134 (16.67)	94 (11.13)	93 (11.61)	52 (5.81)	
Non-Hispanic White	1,104 (75.69)	260 (71.82)	276 (74.20)	268 (76.09)	300 (80.60)	
Other Hispanic	126 (3.30)	32 (3.09)	40 (4.14)	26 (2.99)	28 (2.97)	
Other Race	88 (5.63)	22 (4.61)	21 (7.12)	19 (4.69)	26 (6.03)	
PIR,%	2.62 (0.06)	2.24 (0.10)	2.59 (0.11)	2.79 (0.10)	2.87 (0.11)	<0.001
BMI, km/m^2^	30.43 (0.25)	30.71 (0.53)	31.63 (0.58)	30.41 (0.40)	28.96 (0.37)	<0.001
ALT, u/L	24.44 (0.48)	22.01 (1.16)	23.22 (0.73)	23.26 (0.60)	29.21 (1.21)	<0.001
AST, u/L	25.97 (0.45)	24.66 (1.03)	25.89 (1.25)	24.18 (0.48)	29.08 (0.89)	<0.001
Sodium, mmol/L	139.37 (0.10)	139.24 (0.17)	139.29 (0.18)	139.67 (0.21)	139.28 (0.16)	0.323
Calcium, mmol/L	2.35 (0.00)	2.32 (0.01)	2.36 (0.01)	2.36 (0.01)	2.36 (0.00)	<0.001
Potassium, mmol/L	4.13 (0.01)	4.09 (0.03)	4.17 (0.02)	4.12 (0.03)	4.15 (0.02)	0.170
TC, mg/dl	183.57 (1.39)	177.12 (2.26)	184.70 (3.15)	186.52 (2.55)	185.85 (2.97)	0.026
HDL, mmol/L	1.33 (0.02)	1.31 (0.03)	1.30 (0.03)	1.31 (0.03)	1.41 (0.03)	0.036
Drinking (%)						0.134
Former	601 (29.05)	171 (33.68)	152 (27.18)	150 (28.46)	128 (27.01)	
Never	260 (11.74)	79 (13.50)	74 (13.38)	63 (11.35)	44 (8.74)	
Now	1,042 (59.21)	235 (52.82)	257 (59.44)	253 (60.19)	297 (64.25)	
Smoking (%)						<0.001
Former	764 (39.20)	179 (35.79)	187 (33.95)	185 (40.89)	213 (46.20)	
Never	736 (38.36)	171 (33.73)	215 (47.81)	188 (36.81)	162 (34.81)	
Now	403 (22.44)	135 (30.48)	81 (18.23)	93 (22.30)	94 (18.99)	
Hypertension (%)						0.008
No	446 (25.17)	94 (19.09)	96 (22.11)	120 (27.44)	136 (31.99)	
Yes	1,457 (74.83)	391 (80.91)	387 (77.89)	346 (72.56)	333 (68.01)	
Type 2 DM,%						0.305
No	1,132 (61.26)	280 (61.53)	269 (57.75)	283 (60.34)	300 (65.47)	
Yes	771 (38.74)	205 (38.47)	214 (42.25)	183 (39.66)	169 (34.53)	
All-cause mortality,%						0.006
No	1,081 (66.43)	245 (57.82)	286 (67.47)	282 (71.13)	268 (69.20)	
Yes	822 (33.57)	240 (42.18)	197 (32.53)	184 (28.87)	201 (30.80)	
Cardiovascular mortality,%						<0.001
No	1,620(88.56)	392(82.69)	411(88.39)	403(89.64)	414(93.40)	
Yes	283(11.44)	93(17.31)	72(11.61)	63(10.36)	55(6.60)	

SI, serum iron; PIR, poverty income ratio; BMI, body mass index; ALT, alanine aminotransferase; AST, aspartate transaminase; TC, total serum cholesterol; HDL, high-density lipoprotein; Type 2 DM, type 2 diabetes mellitus.

All values are expressed as a number (%) or mean ± standard deviation.

Table 2 delineates a comparison between participants categorized as survivors and non-survivors based on their all-cause mortality status. Among the 1,903 participants, there were 822 non-survivors and 1,081 survivors. Non-survivors were notably older and exhibited lower PIR and lower BMI compared to survivors. Non-survivors displayed significantly lower levels of ALT, sodium, and SI, along with higher potassium. They also comprised a higher percentage of non-Hispanic white individuals, former-drinkers, never-drinkers, and never-smokers. Additionally, the prevalence of hypertension was notably higher among non-survivors, while the incidence of type 2 DM was decreased compared to survivors.

**Table 2:** Characteristics of the study population according to their mortality.

	Total	Survivor	Non-survivor	*P* value
	(*n* = 1,903)	(*n* = 1,081)	(*n* = 822)	
Age, years	64.29 (0.42)	61.01 (0.49)	70.77 (0.53)	<0.001
Sex (%)				0.108
Female	799 (44.64)	504 (46.41)	295 (41.15)	
Male	1,104 (55.36)	577 (53.59)	527 (58.85)	
Race (%)				0.005
Mexican American	212 (4.10)	133 (4.69)	79 (2.94)	
Non-Hispanic Black	373 (11.28)	241 (12.08)	132 (9.68)	
Non-Hispanic White	1,104 (75.69)	554 (72.83)	550 (81.35)	
Other Hispanic	126 (3.30)	91 (4.06)	35 (1.81)	
Other Race	88 (5.63)	62 (6.34)	26 (4.23)	
PIR,%	2.62 (0.06)	2.75 (0.07)	2.37 (0.07)	<0.001
BMI,%	30.43 (0.25)	31.06 (0.30)	29.18 (0.35)	<0.001
ALT, u/L	24.44 (0.48)	25.09 (0.65)	23.16 (0.67)	0.043
AST, u/L	25.97 (0.45)	25.79 (0.58)	26.33 (0.67)	0.535
Sodium, mmol/L	139.37 (0.10)	139.57 (0.13)	138.97 (0.15)	0.003
Calcium, mmol/L	2.35 (0.00)	2.35 (0.00)	2.35 (0.00)	0.677
Potassium, mmol/L	4.13 (0.01)	4.10 (0.01)	4.19 (0.02)	<0.001
TC, mg/dl	183.57 (1.39)	183.79 (1.84)	183.14 (1.79)	0.795
HDL, mmol/L	1.33 (0.02)	1.33 (0.02)	1.34 (0.02)	0.639
SI, umol/L	15.26 (0.20)	15.57 (0.29)	14.65 (0.25)	0.025
Drinking (%)				<0.001
Former	601 (29.05)	283 (25.08)	318 (36.91)	
Never	260 (11.74)	143 (10.13)	117 (14.92)	
Now	1,042 (59.21)	655 (64.78)	387 (48.17)	
Smoking (%)				0.047
Former	764 (39.20)	393 (36.60)	371 (44.33)	
Never	736 (38.36)	447 (39.88)	289 (35.35)	
Now	403 (22.44)	241 (23.52)	162 (20.32)	
Hypertension (%)				0.003
No	446 (25.17)	283 (28.05)	163 (19.46)	
Yes	1,457 (74.83)	798 (71.95)	659 (80.54)	
Type 2 DM,%				0.002
No	1,132(61.26)	653(64.28)	479(55.31)	
Yes	771(38.74)	428(35.72)	343(44.69)	

SI, serum iron; PIR, poverty income ratio; BMI, body mass index; ALT, alanine aminotransferase; AST, aspartate transaminase; TC, total serum cholesterol; HDL, high-density lipoprotein; Type 2 DM, type 2 diabetes mellitus.

All values are expressed as a number (%) or mean ± standard deviation.

### Association between SI and mortality in CVD patients

**Figure 2:**
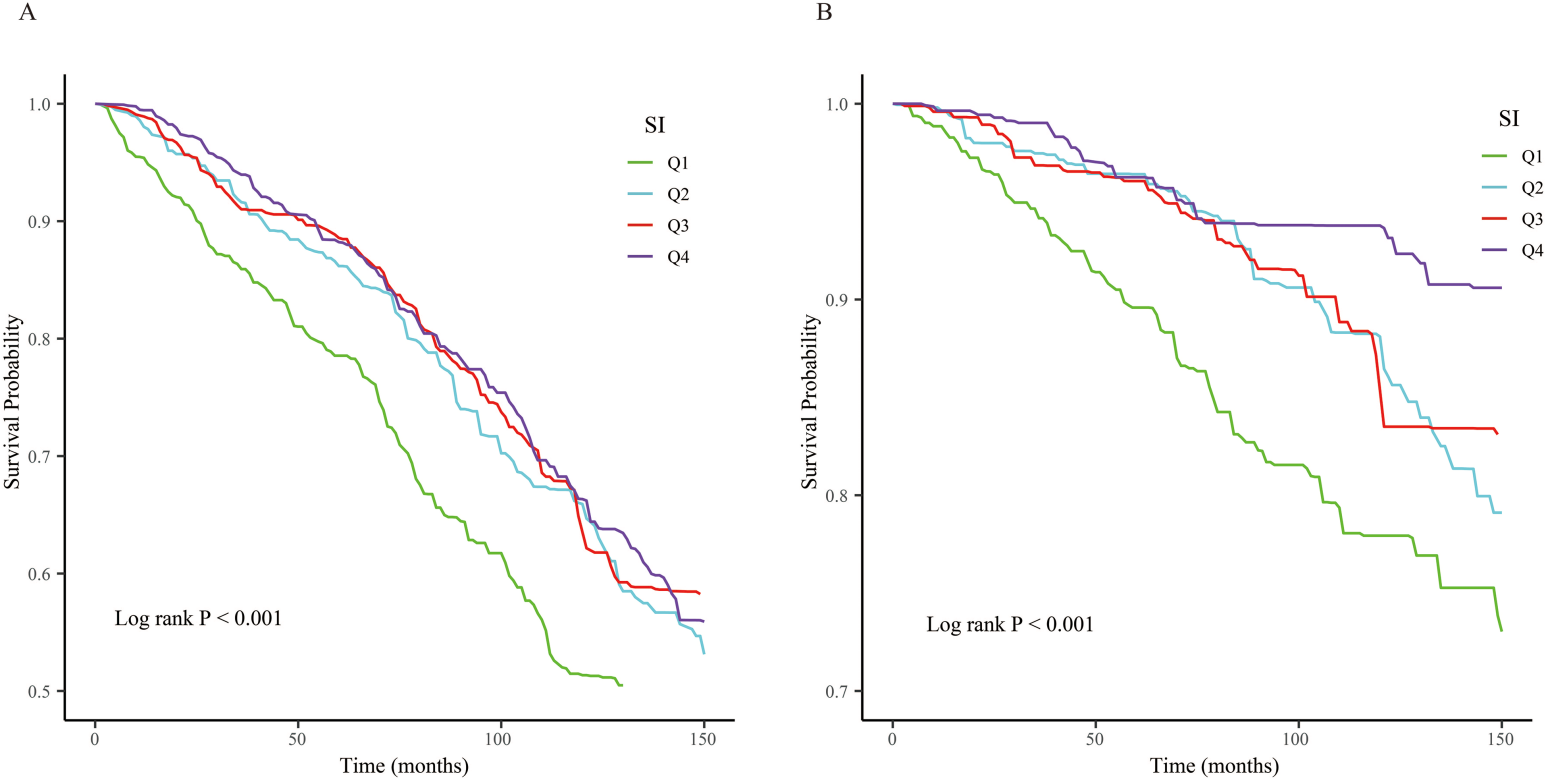
The Kaplan–Meier (KM) survival curves are classified by SI quartiles. **(A)** All-cause mortality. **(B)** Cardiovascular mortality.

KM curves illustrated that when SI was assessed as a categorical variable, patients with the lower SI exhibited higher cardiovascular and all-cause mortality (log-rank *P* < 0.001, all both) (Figure 2).

The association between SI and mortality in CVD patients was analyzed using multivariable Cox regression, as depicted in Table 3. In the unadjusted model, a negative association was observed between SI and all-cause mortality in CVD patients (HR = 0.97, 95% CI: 0.96–0.99). Upon adjusting for relevant covariates, this notable association persisted in the fully adjusted model (HR = 0.97, 95% CI: 0.95–0.99). Furthermore, when stratified by quartiles of SI and analyzed in the fully adjusted Model 2, the adjusted HR along with its 95% CI for SI across the quartiles were as follows: 1.00, 0.69 (95% CI: 0.52–0.92), 0.63 (95% CI: 0.48–0.84), and 0.57 (95% CI: 0.45–0.72).

**Table 3:** Association between SI and all-cause mortality in CVD patients.

	Crude model		Model 1		Model 2	
	HR (95%CI)	*P*	HR (95%CI)	*P*	HR (95%CI)	*P*
SI	0.97 (0.96,0.99)	0.002	0.96 (0.94,0.98)	<0.001	0.97 (0.95,0.99)	<0.001
SI quartiles						
Q1	Ref		Ref		Ref	
Q2	0.71 (0.55,0.91)	0.006	0.63 (0.49,0.82)	<0.001	0.69 (0.52,0.92)	0.010
Q3	0.65 (0.49,0.87)	0.003	0.57 (0.43,0.76)	<0.001	0.63 (0.48,0.84)	0.001
Q4	0.62 (0.49,0.79)	<0.001	0.52 (0.41,0.66)	<0.001	0.57 (0.45,0.72)	<0.001
*P* for trend		<0.001		<0.001		<0.001

Crude model: no adjusted; Model 1: adjusted for age, sex, race, and PIR; Model 2: adjusted for age, sex, race, PIR, drinking, smoking, BMI, hypertension, type 2 DM, ALT, AST, sodium, calcium, potassium, TC, and HDL.

SI, serum iron; PIR, poverty income ratio; BMI, body mass index; ALT, alanine aminotransferase; AST, aspartate transaminase; TC, total serum cholesterol; HDL, high-density lipoprotein; Type 2 DM, type 2 diabetes mellitus; CVD, cardiovascular disease.

Similarly, when using cardiovascular mortality as an outcome indicator, the highest quartile reduced the risk of 74% of deaths compared to the lowest SI quartile (Table 4).

**Table 4:** Association between SI and cardiovascular mortality in CVD patients.

	Crude model		Model 1		Model 2	
	HR (95%CI)	*P*	HR (95%CI)	*P*	HR (95%CI)	*P*
SI	0.94 (0.91,0.96)	<0.001	0.91 (0.88,0.94)	<0.001	0.92 (0.89,0.95)	<0.001
SI quartiles						
Q1	Ref		Ref		Ref	
Q2	0.62 (0.42,0.92)	0.018	0.51 (0.33,0.79)	0.002	0.52 (0.31,0.86)	0.011
Q3	0.58 (0.37,0.90)	0.017	0.47 (0.28,0.77)	0.003	0.50 (0.31,0.82)	0.006
Q4	0.33 (0.21,0.51)	<0.001	0.24 (0.15,0.38)	<0.001	0.26 (0.16,0.43)	<0.001
*P* for trend		<0.001		<0.001		<0.001

Crude model: no adjusted; Model 1: adjusted for age, sex, race, and PIR; Model 2: adjusted for age, sex, race, PIR, drinking, smoking, BMI, hypertension, type 2 DM, ALT, AST, sodium, calcium, potassium, TC, and HDL.

SI: serum iron; PIR, poverty income ratio; BMI, body mass index; ALT, alanine aminotransferase; AST, aspartate transaminase; TC, total serum cholesterol; HDL, high-density lipoprotein; Type 2 DM, type 2 diabetes mellitus; CVD, cardiovascular disease.

### Dose-response relationship

**Figure 3:**
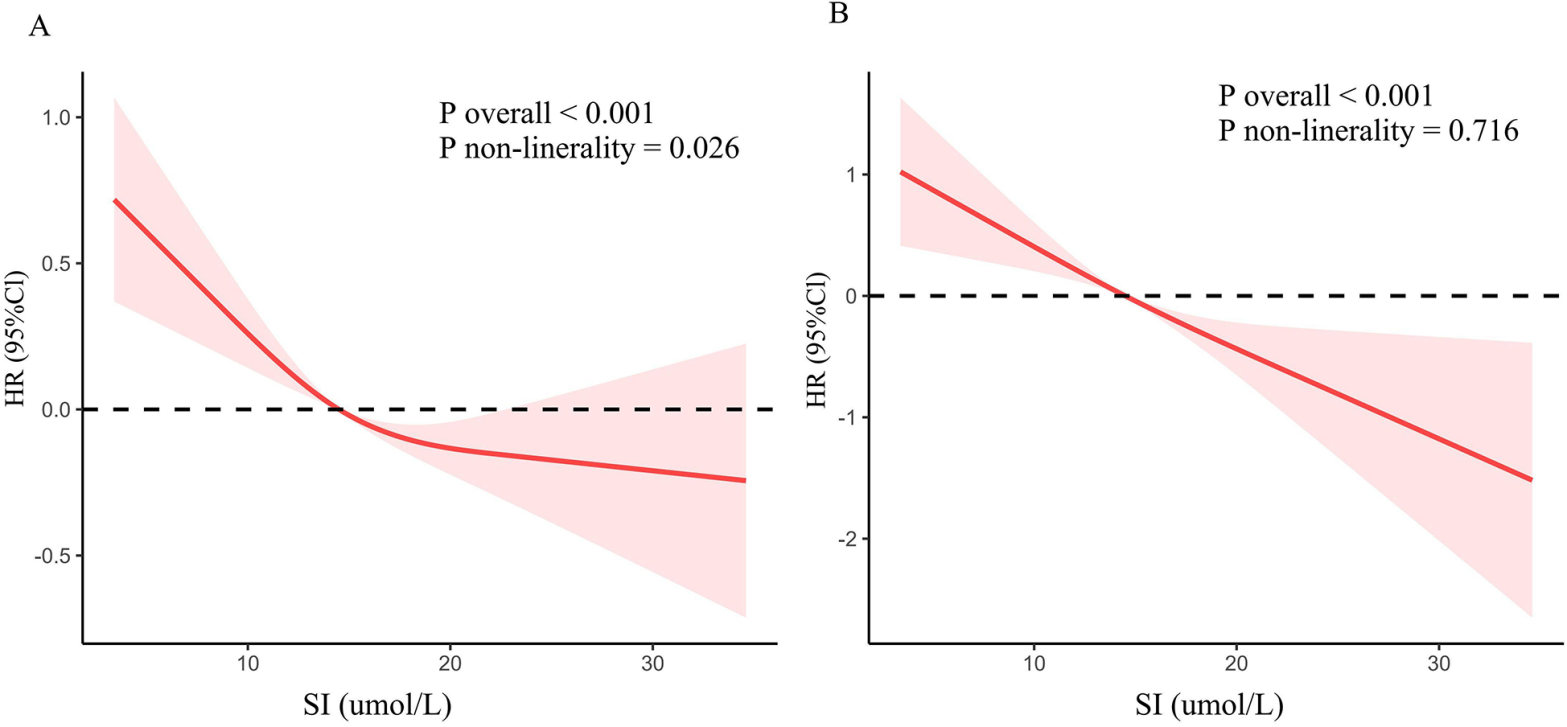
Dose-response association between SI and mortality in CVD population. **(A)** All-cause mortality. **(B)** Cardiovascular mortality.

RCS was utilized to explore the association between SI and cardiovascular and all-cause mortality (Figure 3). The findings revealed an inverted L-shaped relationship between SI and all-cause mortality among patients with CVD (*P* non-linear = 0.026, Figure 3A). Furthermore, a linear negative relationship was identified between SI and cardiovascular mortality (*P* non-linearity = 0.716, Figure 3B).

### Subgroup analysis

**Figure 4:**
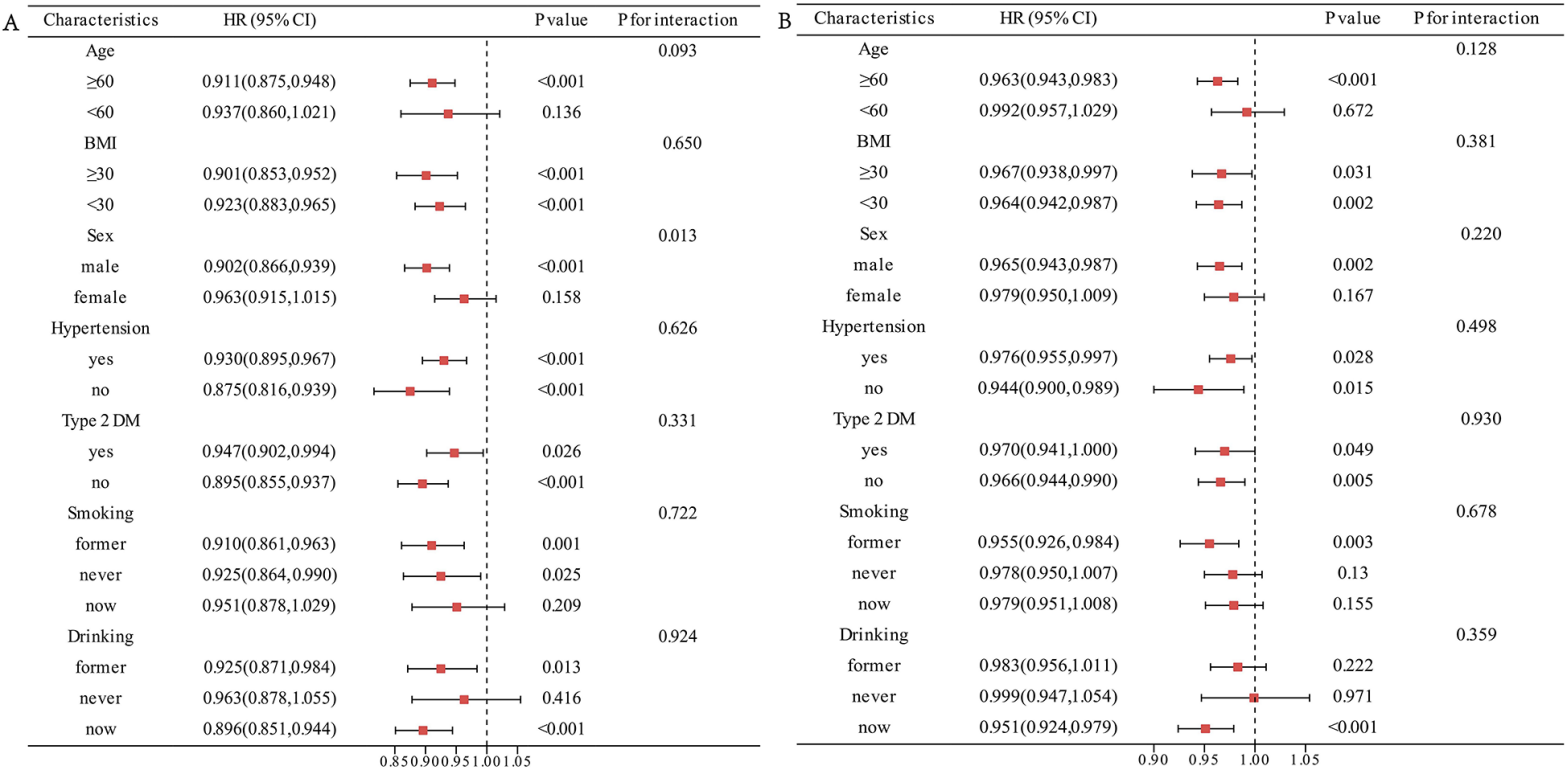
Subgroup analysis of the association between SI and mortality in CVD population. **(A)** All-cause mortality. **(B)** Cardiovascular mortality.

Patients with CVD were stratified into distinct subgroups based on age, sex, BMI, hypertension, type 2 DM, smoking, and drinking. The impact of SI levels on all-cause and cardiovascular mortality among different characteristics of CVD patients was examined, and the results were depicted in forest plots (Figure 4). The outcomes indicated a significant association between SI and all-cause mortality within specific subgroups, including males, age ≥ 60, BMI ≥ 30 kg/m^2^, hypertension, type 2 DM, former smokers, and drinkers. Interaction analysis unveiled that the relationship between SI levels and all-cause mortality in CVD patients did not exhibit significant variations across the various subgroups (interaction *P* > 0.05) (Figure 4A).

Similarly, the negative association of SI with cardiovascular mortality in males, age ≥ 60, BMI < 30 kg/m^2^, hypertension, type 2 DM, never-smokers, former smokers, and drinkers. In addition, interaction analysis revealed that the relationship between SI levels and cardiovascular mortality differed among sex subgroups (interaction *P* < 0.001) (Figure 4B).

## Discussion

Our findings suggest a significant negative association between SI and all-cause and cardiovascular mortality in CVD patients, even after adjusting for potential confounders. In addition, RCS curve analysis highlighted a nonlinear negative association between SI levels and all-cause mortality and a linear negative association with cardiovascular mortality. Subgroup analyses and interaction tests further clarified that the negative association between SI levels and all-cause mortality was more significant among males, age ≥60, BMI ≥30 kg/m^2^, hypertension, type 2 DM, former smokers, and drinkers. On the other hand, the negative association between SI levels and cardiovascular mortality was stronger in males, age ≥ 60, BMI < 30 kg/m^2^, hypertension, type 2 DM, never smokers, former drinkers, and drinkers.

Iron, an essential mineral nutrient, plays a pivotal role in hemoglobin synthesis, iron-dependent catalytic reactions, DNA synthesis, and mitochondrial respiration (14). SI levels have been closely linked to various diseases, displaying differing associations across diverse medical conditions. Notably, in sepsis patients, a large-scale study revealed a positive correlation between SI levels and 90-day mortality, with higher quartiles of SI reflecting elevated 90-day mortality (15). Likewise, in liver-related studies, SI levels were positively associated with the mortality of chronic liver disease (16, 17), while in patients with acute kidney injury, high SI levels were correlated with increased 28-day and 90-day mortality (18). Conversely, in certain diseases, SI levels exhibit an inverse relationship with mortality. For example, lower total body iron levels were found to elevate the risk of death in hemodialysis patients in a study from Japan (19). Studies have also demonstrated the importance of SI levels in infectious diseases, for example, reduced SI levels were observed in patients with severe COVID-19 infection (20). Similarly, in H7N9 infections, reduced SI levels, especially in patients who died, indicate the severity of the disease (21). Additionally, a study focusing on cancer patients revealed that individuals with elevated SI levels exhibited a reduced risk of mortality from oral cancer (22). An investigation conducted in China identified low SI levels as an independent risk factor for in-hospital mortality among critically ill patients (23). The reason for the different associations may be due to the dichotomy of SI (24). Firstly, most of the body's iron is contained in the hemoglobin (Hb) of erythrocytes: iron deficiency leads to anaemia due to reduced Hb synthesis and erythropoiesis. In addition, severe hypoferremia affects the reduced synthesis of iron-containing enzymes and proteins essential for cellular function; thus, low SI is associated with increased mortality from diseases such as CVD. On the other hand, the chemical reactivity of iron makes it potentially very toxic: ferrous iron (Fe^2+^) is unstable under aerobic conditions and triggers the Fenton reaction, which produces reactive oxygen species that can damage proteins, membranes, and DNA In order to avoid the potentially damaging effects of iron, this metal is always conjugated to proteins to facilitate their uptake and transport, as well as their storage in the cell. In the human body, iron-regulated proteins are mainly found in the liver, and when dysfunctional, iron overload leads to cell and tissue damage due to the presence of unconjugated iron, which ultimately leads to damage to cell membrane structures and triggers cell death (25, 26). Thus, excessive SI leads to increased mortality in liver-related and other diseases.

Previous research has extensively demonstrated the close relationship between SI levels and CVD. Iron deficiency serves as a noticeable indicator in heart failure cases, with a significant proportion of heart failure patients exhibiting iron deficiency, ranging from 47% to 68% in individuals with chronic heart failure. Moreover, the severity of heart failure is positively correlated with the likelihood of iron deficiency (27). Iron deficiency within myocardial cells can impede mitochondrial respiration and the ability to adapt to acute and chronic increases in workload. Notably, iron supplementation has been shown to enhance heart failure management and prevent its onset (6, 28, 29, 30). Additionally, iron deficiency is prevalent among individuals with pulmonary arterial hypertension, and intravenous iron supplementation has been found to enhance patients' quality of life and diminish the risk of hospitalization (31). Iron deficiency anemia accounts for approximately 50% of anemic cases and is recognized as a risk factor for ischemic stroke (32, 33). Moreover, iron deficiency has been associated with an augmented risk of platelet aggregation and thrombus formation (34, 35), as well as a heightened risk of developing coronary heart disease (36). Studies targeting individuals with chronic kidney disease have shown that iron supplementation in iron-deficient patients can reduce the risk of cardiovascular disease (37). These findings align with our results, indicating that lower SI levels are linked to increased cardiovascular mortality and all-cause mortality among patients with cardiovascular conditions.

Our study is subject to certain limitations that warrant consideration. Firstly, the retrospective observational cohort study design restricts our ability to establish a definitive causal relationship between SI and mortality among CVD patients, as we can only infer associations rather than prove causation. Secondly, our analysis relies solely on SI data from the NHANES database, which lacks additional details such as iron binding capacity, transferrin saturation, and other related parameters. Lastly, the utilization of a single-point measurement technique for SI assessment may not fully capture the dynamic fluctuations in SI concentrations, with diurnal variations in SI levels noted. As such, further research endeavors are warranted to delve deeper into this area and address these limitations for a more comprehensive understanding of the associations between SI and mortality in CVD patients.

## Conclusion

In our study, we found an inverted L-shaped association of SI with all-cause mortality and a linear negative association with cardiovascular mortality in patients with CVD, which provides a valuable reference to guide the management of SI in patients with CVD.

## Data Availability

The raw data supporting the conclusions of this article will be made available by the authors, without undue reservation.
