# Supplementary material for: Association of serum iron with all-cause mortality and cardiovascular mortality in the cardiovascular patients: a retrospective cohort study based on the NHANES 1999–2018
Source: Front Cardiovasc Med. 2024 Dec 10;11:1414792. doi: 10.3389/fcvm.2024.1414792 (PMC11666567; doi:10.3389/fcvm.2024.1414792)
Supplement: Supplementary file 1 [file Table1.docx]

| **Supplementary Table 1. Baseline characteristics between eligible patients and those excluded patients** | | | | |
| --- | --- | --- | --- | --- |
|  | **Total**  **n=5489** | **Excluded**  **N=3586** | **Eligible**  **N=1903** | **P value** |
|  |  |  |  |  |
| Age, years | 66.91(13.05) | 67.24(13.23) | 66.28(12.68) | 0.010 |
| Sex, N(%) |  |  |  | 0.151 |
| Female | 2377(43.30) | 1578(44.00) | 799(41.99) |  |
| Male | 3112(56.70) | 2008(56.00) | 1104(58.01) |  |
| Race, N(%) |  |  |  | 0.014 |
| Mexican American | 643(11.71) | 431(12.02) | 212(11.14) |  |
| Non-Hispanic Black | 1110(20.22) | 737(20.55) | 373(19.60) |  |
| Non-Hispanic White | 3082(56.15) | 1978(55.16) | 1104(58.01) |  |
| Other Hispanic | 332(6.05) | 206(5.74) | 126(6.62) |  |
| Other Race | 322(5.87) | 234(6.53) | 88(4.62) |  |
| PIR | 2.21(1.47) | 2.15(1.45) | 2.31(1.51) | <0.001 |
| BMI, km/m^2^ | 30.08(6.99) | 30.07(7.02) | 30.09(6.95) | 0.951 |
| ALT, u/L | 23.33(25.63) | 22.94(29.26) | 24.06(16.84) | 0.126 |
| AST, u/L | 25.50(18.68) | 25.18(16.07) | 26.10(22.79) | 0.083 |
| Sodium, mmol/L | 139.36(2.88) | 139.36(3.02) | 139.35(2.61) | 0.868 |
| Calcium, mmol/L | 2.35(0.10) | 2.35(0.11) | 2.35(0.10) | 0.322 |
| Potassium, mmol/L | 4.12(0.43) | 4.12(0.43) | 4.14(0.41) | 0.057 |
| SI, umol/L | 14.31(5.93) | 13.86(5.89) | 15.16(5.92) | <0.001 |
| TC, mg/dL | 185.30(45.88) | 185.70(45.70) | 184.53(46.22) | 0.371 |
| HDL, mmol/L | 1.30(0.41) | 1.29(0.41) | 1.31(0.41) | 0.056 |
| Drinking, N(%) |  |  |  | 0.002 |
| Former | 1622(33.21) | 1021(34.25) | 601(31.58) |  |
| Never | 738(15.11) | 478(16.03) | 260(13.66) |  |
| Now | 2524(51.68) | 1482(49.71) | 1042(54.76) |  |
| Smoking, N(%) |  |  |  | 0.170 |
| Former | 2250(41.01) | 1486(41.47) | 764(40.15) |  |
| Never | 2150(39.19) | 1414(39.46) | 736(38.68) |  |
| Now | 1086(19.80) | 683(19.06) | 403(21.18) |  |
| Hypertension, N(%) |  |  |  | 0.219 |
| No | 1234(22.49) | 788(21.98) | 446(23.44) |  |
| Yes | 4254(77.51) | 2797(78.02) | 1457(76.56) |  |
| Type 2 DM, N(%) |  |  |  | 0.437 |
| No | 3299(60.19) | 2167(60.56) | 1132(59.49) |  |
| Yes | 2182(39.81) | 1411(39.44) | 771(40.51) |  |
| All-cause mortality, N(%) |  |  |  | 0.178 |
| No | 3050(55.57) | 1969(54.91) | 1081(56.81) |  |
| Yes | 2439(44.43) | 1617(45.09) | 822(43.19) |  |
| Cardiovascular mortality, N(%) |  |  |  | 0.686 |
| No | 4658(84.86) | 3038(84.72) | 1620(85.13) |  |
| Yes | 831(15.14) | 548(15.28) | 283(14.87) |  |
